# A curious case of pulmonary hypertension in a child

**DOI:** 10.1186/s43044-022-00294-6

**Published:** 2022-08-05

**Authors:** A. Shaheer Ahmed, Gaurav Kumar Divani, Shivank Gupta

**Affiliations:** Department of Cardiology, Vardhman Mahavir Medical College and Safdarjung Hospital, 7th floor, Super speciality block, New Delhi, 110029 India

**Keywords:** Pulmonary hypertension, Chronic constrictive pericarditis, Tuberculous pericarditis

## Abstract

**Background:**

Pulmonary hypertension in young children can be due to a myriad of conditions. Few aetiologies of pulmonary hypertension are potentially reversible. An extensive workup for the cause of pulmonary hypertension is a must before attributing it to idiopathic pulmonary hypertension. We describe an uncommon aetiology of pulmonary hypertension in a young boy.

**Case presentation:**

A 12-year-old child, with past history of tubercular pleural effusion, presented with dyspnoea on exertion and easy fatiguability for 2 years. He was evaluated elsewhere and was being treated as primary pulmonary hypertension with pulmonary vasodilators. The child was revaluated since the clinical features were not completely favouring the diagnosis. On detailed evaluation, a diagnosis of constrictive pericarditis was made. He was referred for pericardiectomy.

**Conclusions:**

Constrictive pericarditis presenting with severe pulmonary hypertension without congestive symptoms is very rare. In patients presenting with pulmonary hypertension, always look for a reversible cause before labeling them as idiopathic PAH.

**Supplementary Information:**

The online version contains supplementary material available at 10.1186/s43044-022-00294-6.

## Background

Pulmonary hypertension in young children can be due to a myriad of conditions. Few aetiologies of pulmonary hypertension are potentially reversible. An extensive workup for the cause of pulmonary hypertension is a must before attributing it to idiopathic pulmonary hypertension. Pulmonary hypertension is relatively uncommon in chronic constrictive pericarditis, especially in children. We describe an uncommon aetiology of pulmonary hypertension in a young boy.

## Case presentation

A 12-year-old child presented with history of dyspnoea on exertion NYHA class II and easy fatiguability for 2 years. He did not have any history of abdominal distention or pedal oedema. He had past history of tubercular pleural effusion 3 years back and had completed 6-month antitubercular therapy. He was evaluated elsewhere and was being treated as primary pulmonary arterial hypertension with pulmonary vasodilators and diuretics. He was referred to a higher centre for further management. Since the clinical features were not completely favouring the diagnosis, we re-evaluated the child.

**Fig. 1:**
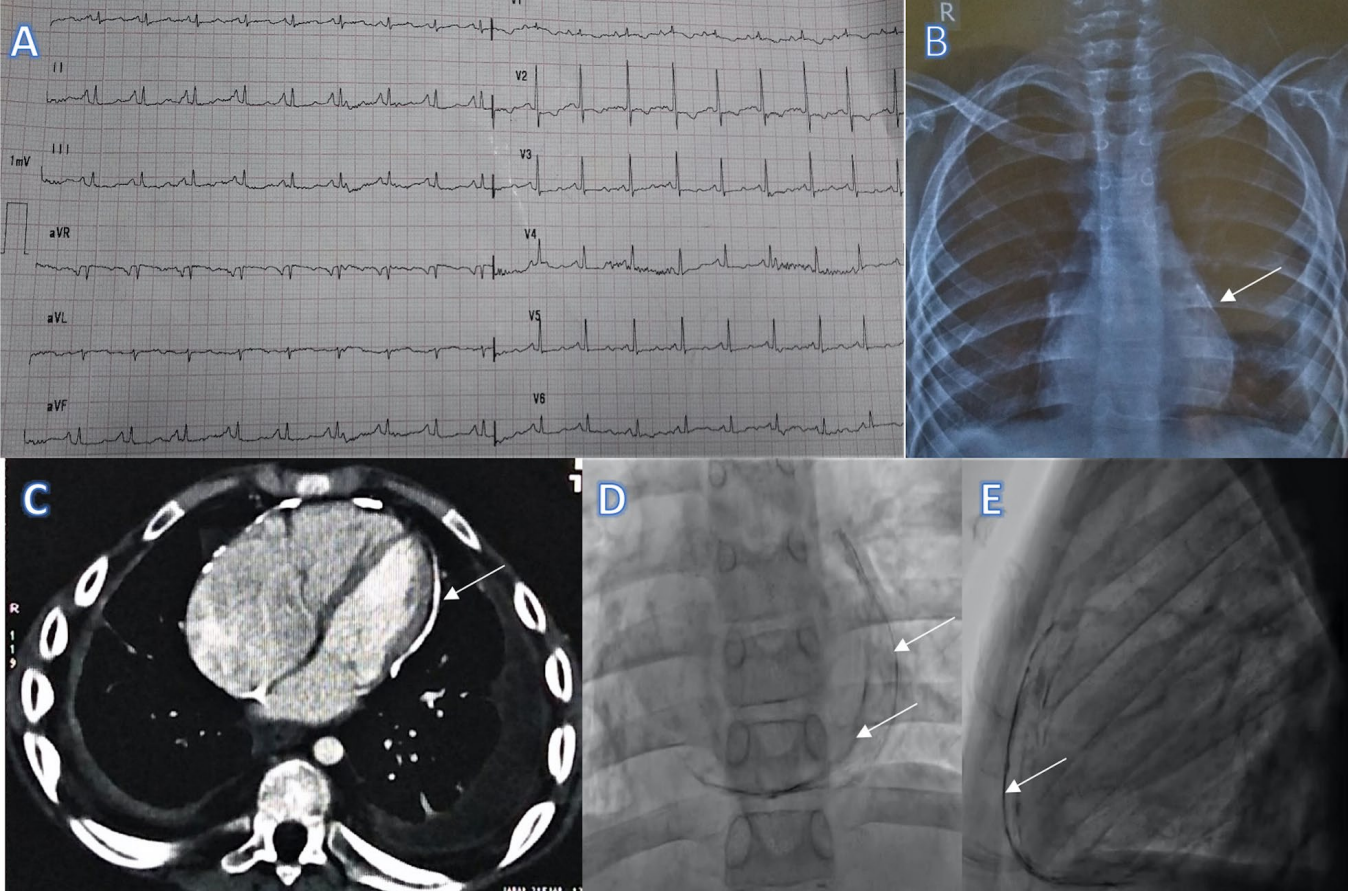
**A** Electrocardiogram showing low-voltage QRS complexes in limb leads, left atrial enlargement and non-specific repolarisation changes. **B** Chest X ray PA view showing features of pulmonary venous hypertension and pericardial calcification (arrow) **C** CT scan image showing pericardial calcification **D** Cine fluoroscopy in AP view showing pericardial calcification **E** Cine fluoroscopy in lateral view showing pericardial calcification

**Fig. 2:**
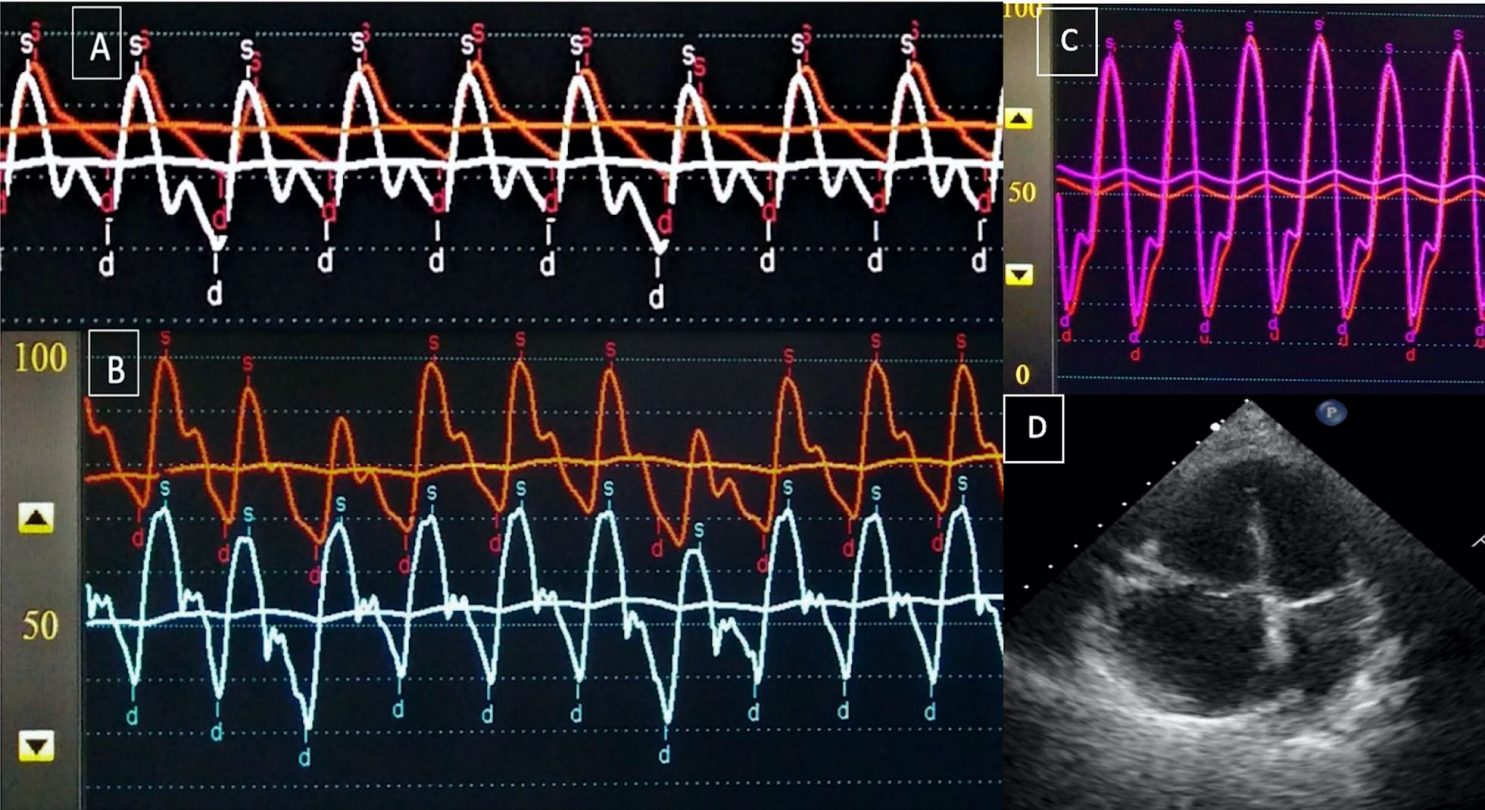
**A** Simultaneous aorta and pulmonary artery pressure trace at baseline **B** Simultaneous aorta and pulmonary artery pressure trace after 100% oxygen administration **C** Ventricular pressure tracing showing prominent rapid filling wave and equalisation of ventricular diastolic pressure **D** Echocardiography in apical four chamber view showing dilated right atrium and ventricle

On examination, the jugular venous pressure was elevated, but the waveforms were not clearly discernible due to tachycardia. On auscultation, P2 was loud and there were no added sounds. Electrocardiogram (ECG) revealed bi-atrial enlargement, low-voltage QRS complexes and non-specific ST-T changes. These ECG findings were suggestive of either constrictive pericarditis or restrictive cardiomyopathy and not in favour of pulmonary hypertension. Chest X-ray showed left-sided loculated pleural effusion and features of pulmonary venous hypertension. On having a closer look, pericardial calcification was evident. The pericardial calcification was more prominent in cine fluoroscopy (Fig. 1). He had an average transthoracic echocardiographic window. The most glaring finding in the echocardiography was dilated right atrium and ventricle with right ventricular dysfunction. Right ventricular systolic pressure was 60 mmHg + RAP. However, on detailed examination features of constrictive pericarditis like septal bounce, annulus reversus and paradoxus, E/A = 2:1 and expiratory hepatic vein reversal were present (Additional files 1, 2). Contrast-enhanced CT revealed thickened pericardium (5 mm) with calcification. Patient was taken up for cardiac catheterisation after tapping the left pleural effusion, as it might influence the PVRi calculation. There was elevation and equalisation of diastolic pressures in all the cardiac chambers. Left ventricular rapid filling wave and square root pattern were seen in the ventricular pressure trace (Fig. 2). However, the ventricular interdependence was not prominent, probably because of a dilated and dysfunctional right ventricle. Baseline pulmonary artery pressure was 87/46/63 mmHg (sys/dias/mean) with corresponding aortic pressure of 89/63/74 mmHg, and PVRi was 17.1 woods unit. The cardiac index was 1.9 L/mm2, and PVR/SVR ratio was 0.7. Subsequently, 100% oxygen for 10 min was administered and repeated the measurements. Post-oxygen inhalation, the pulmonary artery pressure reduced to 70/37/54 mmHg with corresponding aortic pressure of 97/68/80 mmHg. The PVRi ratio and PVR/SVR ratio post-oxygen administration were 13.6 woods unit and 0.4, respectively. There was more than 20% fall in PVRi and PVR/SVR ratio in response to oxygen, which signifies reactive pulmonary hypertension. Sildenafil and furosemide were continued. Patient was referred for pericardiectomy.

Constrictive pericarditis in paediatric age group is relatively uncommon. Even in those with constrictive pericarditis, presence of pericardial calcification is even more uncommon. Pericardial calcification is more frequently seen in constrictive pericarditis of tubercular aetiology [1]. Overall, pericardial calcification is evident in around 25–30% in case CCP. This estimate is much lower in paediatric age group. Pericardial calcification is mostly seen in the atrioventricular groove and anterior to the right ventricle, which is more obvious in lateral and oblique chest radiograph [2]. Pulmonary artery pressure in constrictive pericarditis in usually less than 50 mmHg. However, in around 5–20% of the cases, pulmonary hypertension may be present [3, 4]. Pulmonary hypertension is more prevalent in those who received radiation therapy and those with past history of cardiac surgery [3]. The probable mechanisms contemplated for pulmonary hypertension in constrictive pericarditis include myocardial involvement, predominant left-sided constriction, constriction of left AV groove leading to LV inflow obstruction and epicardial coronary narrowing by thickened pericardium [5]. Treating such patients with pulmonary vasodilators might lead to worsening of symptoms in some patients [6]. Although in our case there was some improvement in symptoms, one should be vigilant while initiating vasodilators. Pericardiectomy in the presence of pulmonary hypertension carries a higher risk. In the presence of severe PAH, stripping of pericardium over the left ventricle should be done first. Such patients might require iNO ventilation and pulmonary vasodilators during the perioperative period. Pulmonary vasodilators and pericardiectomy help to reverse pulmonary hypertension in such cases [7].

Constrictive pericarditis presenting as pulmonary hypertension, without predominant congestive symptoms, is very rare. Constrictive pericarditis seldom seen in children, and pericardial calcification is even rarer in this age group. Absence of congestive symptoms, past history of pleural effusion, presence of predominant RA and RV dilatation with pulmonary hypertension masqueraded the actual diagnosis. This case demonstrates that in this era of diminishing clinical skills and increased dependence of echocardiography for cardiac diagnosis, an eagle-eyed interpretation of simple, time tested investigations like chest X-ray and electrocardiogram provide crucial details for arriving at correct diagnosis. Involvement of left more than the right ventricle might lead to pulmonary hypertension in constrictive pericarditis. Presence of RV dilatation and dysfunction might have attenuated ventricular interdependence in our case.

## Conclusions

In patients presenting with pulmonary hypertension, always look for a reversible cause before writing him/her off as a case of idiopathic PAH, which can bring a huge change in their lives.

## Supplementary Information

**Additional file 1**: Echocardiography in apical four chamber view showing dilated right atrium and ventricle and septal bounce.

**Additional file 2**: Fig. S1. **A** continuous wave doppler across the tricuspid valve **B** Hepatic vein doppler showing expiratory flow reversal **C** Medial mitral annulus tissue doppler showing medial E' velocity and annulus paradoxus **D** M mode across IVC.

## Data Availability

The data are available for sharing.

## Abbreviations

NYHA: New York Heart Association
ECG: Electrocardiogram
RAP: Right atrial pressure
PVRi: Pulmonary vascular resistance indexed
SVR: Systemic vascular resistance
CCP: Chronic constrictive pericarditis
LV: Left ventricle
NO: Nitric oxide
AV: Atrioventricular
RA: Right atrium
RV: Right ventricle
PAH: Pulmonary arterial hypertension
