# Supplementary figures and images for: A curious case of pulmonary hypertension in a child
Source: Egypt Heart J. 2022 Aug 5;74:58. doi: 10.1186/s43044-022-00294-6 (PMC9356119; doi:10.1186/s43044-022-00294-6)

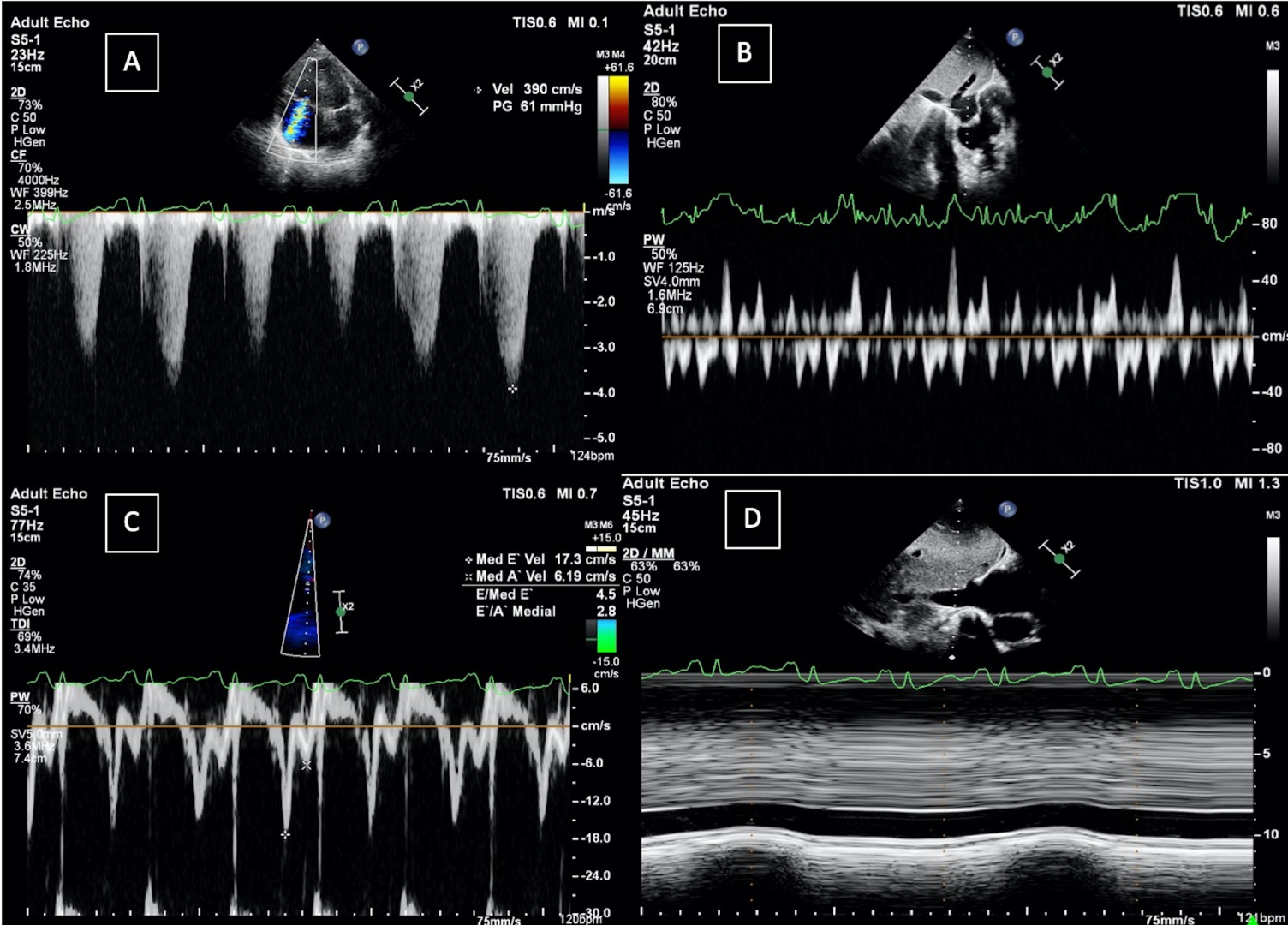

Supplement: Supplementary file 2 — Additional file 2: Fig. S1. A continuous wave doppler across the tricuspid valve B Hepatic vein doppler showing expiratory flow reversal C Medial mitral annulus tissue doppler showing medial E' velocity and annulus paradoxus D M mode across IVC. [file 43044_2022_294_MOESM2_ESM.jpg]
